# The effect of targeting Tie2 on hemorrhagic shock-induced renal perfusion disturbances in rats

**DOI:** 10.1186/s40635-021-00389-5

**Published:** 2021-05-17

**Authors:** Anoek L. I. van Leeuwen, Nicole A. M. Dekker, Paul Van Slyke, Esther de Groot, Marc G. Vervloet, Joris J. T. H. Roelofs, Matijs van Meurs, Charissa E. van den Brom

**Affiliations:** 1 Department of Anesthesiology, Experimental Laboratory for Vital Signs, Amsterdam Cardiovascular Sciences, Amsterdam UMC, Vrije Universiteit, De Boelelaan 1117, 1081 HV Amsterdam, The Netherlands; 2 Department of Physiology, Amsterdam Cardiovascular Sciences, Amsterdam UMC, Vrije Universiteit, Amsterdam, The Netherlands; 3 Department of Cardiothoracic Surgery, Amsterdam Cardiovascular Sciences, Amsterdam UMC, Vrije Universiteit, Amsterdam, The Netherlands; 4 Vasomune Therapeutics, Toronto, Canada; 5 Department of Nephrology, Amsterdam Cardiovascular Sciences, VU University Medical Center, Amsterdam, the Netherlands; 6 Department of Pathology, Amsterdam Cardiovascular Sciences, Academic Medical Center, University of Amsterdam, Amsterdam, Netherlands; 7 Department of Pathology and Medical Biology, Medical Biology Section, University Medical Center Groningen, Groningen, The Netherlands; 8 Department of Critical Care Medicine, University Medical Center Groningen, Groningen, The Netherlands; 9 Department of Intensive Care, Amsterdam UMC, University of Amsterdam, Amsterdam, The Netherlands

**Keywords:** Hemorrhage, Acute kidney injury, Contrast-enhanced ultrasonography, Microcirculatory perfusion, Vascular leakage, Endothelium

## Abstract

**Background:**

Hemorrhagic shock is associated with acute kidney injury and increased mortality. Targeting the endothelial angiopoietin/Tie2 system, which regulates endothelial permeability, previously reduced hemorrhagic shock-induced vascular leakage. We hypothesized that as a consequence of vascular leakage, renal perfusion and function is impaired and that activating Tie2 restores renal perfusion and function.

**Methods:**

Rats underwent 1 h of hemorrhagic shock and were treated with either vasculotide or PBS as control, followed by fluid resuscitation for 4 h. Microcirculatory perfusion was measured in the renal cortex and cremaster muscle using contrast echography and intravital microscopy, respectively. Changes in the angiopoietin/Tie2 system and renal injury markers were measured in plasma and on protein and mRNA level in renal tissue. Renal edema formation was determined by wet/dry weight ratios and renal structure by histological analysis.

**Results:**

Hemorrhagic shock significantly decreased renal perfusion (240 ± 138 to 51 ± 40, *p* < 0.0001) and cremaster perfusion (12 ± 2 to 5 ± 2 perfused vessels, *p* < 0.0001) compared to baseline values. Fluid resuscitation partially restored both perfusion parameters, but both remained below baseline values (renal perfusion 120 ± 58, *p* = 0.08, cremaster perfusion 7 ± 2 perfused vessels, *p* < 0.0001 compared to baseline). Hemorrhagic shock increased circulating angiopoietin-1 (*p* < 0.0001), angiopoietin-2 (*p* < 0.0001) and soluble Tie2 (*p* = 0.05), of which angiopoietin-2 elevation was associated with renal edema formation (r = 0.81, *p* < 0.0001). Hemorrhagic shock induced renal injury, as assessed by increased levels of plasma neutrophil gelatinase-associated lipocalin (NGAL: *p* < 0.05), kidney injury marker-1 (KIM-1; *p* < 0.01) and creatinine (*p* < 0.05). Vasculotide did not improve renal perfusion (*p* > 0.9 at all time points) or reduce renal injury (NGAL *p* = 0.26, KIM-1 *p* = 0.78, creatinine *p* > 0.9, renal edema *p* = 0.08), but temporarily improved cremaster perfusion at 3 h following start of fluid resuscitation compared to untreated rats (resuscitation + 3 h: 11 ± 3 vs 8 ± 3 perfused vessels, *p* < 0.05).

**Conclusion:**

Hemorrhagic shock-induced renal impairment cannot be restored by standard fluid resuscitation, nor by activation of Tie2. Future treatment strategies should focus on reducing angiopoietin-2 levels or on activating Tie2 via an alternative strategy.

**Supplementary Information:**

The online version contains supplementary material available at 10.1186/s40635-021-00389-5.

## Introduction

Hemorrhagic shock is associated with increased mortality and organ failure [1]. In particular, acute kidney injury (AKI) is a major complication following hemorrhagic shock and contributes to prolonged hospital stay and increased mortality [2]. The incidence of AKI following hemorrhagic shock is reported up to 25%, despite early control of bleeding and adequate fluid resuscitation [3]. Unfortunately, effective additional drug treatment strategies to reduce the incidence of AKI are lacking.

Sublingual microcirculatory perfusion is disturbed immediately following hemorrhagic shock [1, 4] and prolonged disturbances in microcirculatory perfusion are associated with multiple organ failure [1]. The renal vasculature is particularly vulnerable for acute blood flow deficits due to its high energy demand [5]. As a consequence of disturbed renal perfusion, oxygen delivery is impaired [6], which leads to disturbed renal function [7]. Although the general pathophysiology of AKI is well described, studies investigating the course of perfusion following hemorrhagic shock are limited [8, 9].

One of the underlying mechanisms that compromises microcirculatory perfusion is increased endothelial permeability, which results in leakage of fluid to the interstitium and edema formation. In rats, we previously showed that hemorrhagic shock-induced renal vascular leakage coincided with microcirculatory perfusion disturbances in the cremaster muscle [10]. Additionally, our group showed that plasma from traumatic hemorrhagic shock patients induced endothelial hyperpermeability, which associated with sublingual microcirculatory perfusion disturbances [11]. An important regulator of endothelial permeability is the angiopoietin/Tie2 system [12]. In healthy physiology, angiopoietin-1 binds to the tyrosine kinase receptor Tie2, resulting in phosphorylation of Tie2 and thereby maintaining endothelial barrier function [12]. Following acute inflammation, as seen during hemorrhagic shock, angiopoietin-2 is released from the Weibel–Palade bodies, which antagonistically binds to and inhibits phosphorylation of Tie2, thereby increasing endothelial permeability [13]. Increased levels of plasma angiopoietin-2 have also been associated with hypoperfusion [14] and increased mortality following trauma [15], and with the development of AKI following cardiac surgery in patients [16]. In rodents, hemorrhagic shock reduced the expression of Tie2 [10, 17] and direct suppression of Tie2 in mice resulted in vascular leakage [18]. Due to its key function in the regulation of endothelial barrier function, targeting Tie2 has been proposed as a promising strategy to improve outcome of critically ill patients [12]. In line with this hypothesis, we previously reported that targeting Tie2 with vasculotide, a Tie2 agonist, restored cremaster perfusion and reduced microvascular leakage in a rat model of hemorrhagic shock and fluid resuscitation [10]. Additionally, in a murine model of ischemia–reperfusion injury, treatment with vasculotide protected renal perfusion, reduced renal edema formation and improved survival [19], proposing a possible application for vasculotide in the treatment of AKI following hemorrhagic shock.

In the present study, we therefore investigated the effect of hemorrhagic shock and fluid resuscitation on renal perfusion and function and studied whether activation of Tie2, in addition to fluid resuscitation, restores renal perfusion and function following hemorrhagic shock and fluid resuscitation in rats.

## Methods

### Animals and experimental set-up

**Fig. 1:**
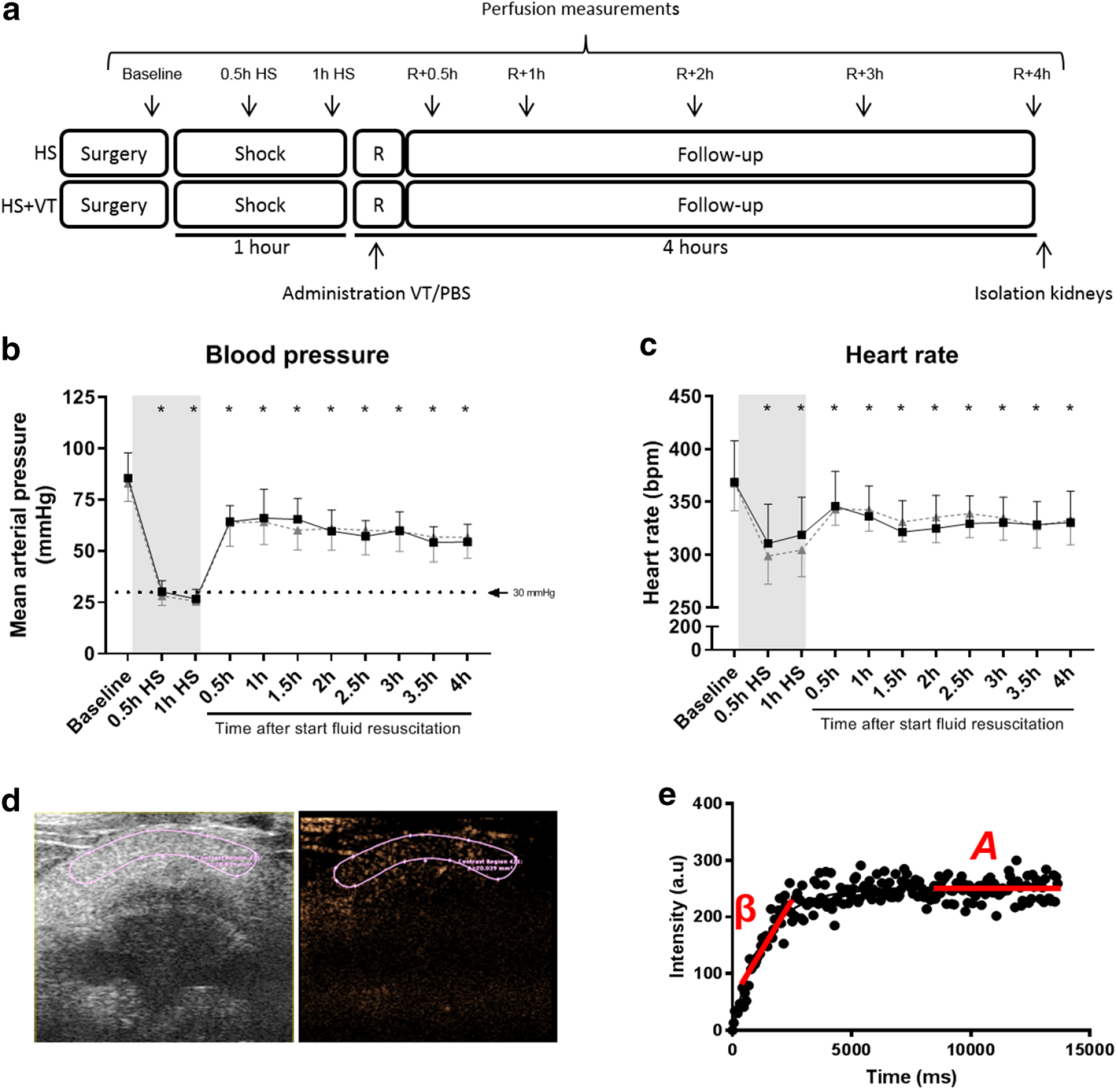
Experimental protocol. Schematic overview of experimental protocol (**a**). Hemorrhagic shock was induced by pressure-controlled blood withdrawal, and mean arterial pressure (MAP) was maintained for 1 h at 30 mmHg (shock). After 1 h of shock, animals were resuscitated with fluids (R), which was paralleled by administration of vasculotide (VT) as treatment or PBS as control, and monitored for 4 consecutive hours (**b**, **c**). Renal and cremaster perfusion measurements were performed directly after the surgical preparation (baseline), 30 min after shock induction (0.5 h HS), 1 h after shock induction (1 h HS), and 30 min (R + 0.5 h), 1 h (R + 1 h), 2 h (R + 2 h), 3 h (R + 3 h) and 4 h (R + 4 h) after start of fluid resuscitation. Plasma was collected at baseline and before killing (4 h after starting fluid resuscitation). Rats were killed and kidneys were isolated for additional molecular analyses and determination of edema formation. **d**, **e** An example of renal perfusion analysis. Regions of interest were drawn in the cortex of the kidney (**d**). The estimate of perfusion was calculated as the product of microvascular blood volume *A* and microvascular filling velocity *β* (**e**). Two-way ANOVA with Bonferroni post hoc analyses, *P < 0.05 HS group compared to baseline; ^#^P < 0.05 HS + VT vs. HS group. Data represent mean ± SD, n = 13

All procedures were approved by the Institutional Animal Care and Use Committee of the VU University, the Netherlands (Animal welfare number: ANES 13-03A2), and conducted following the European Convention for the Protection of Vertebrate Animals used for Experimental and Other Scientific Purposes and the ARRIVE guidelines on animal research [20]. Male Wistar rats of 350–400 g (Charles River Laboratories, Brussels, Belgium) were housed in a temperature-controlled room (12/12 h day/night cycle, 20–23 °C, 40–60% humidity) with food and water ad libitum. Rats underwent hemorrhagic shock with fluid resuscitation and were randomized and treated blindly with vasculotide (Vasomune Therapeutics, Toronto, Canada) (n = 14) or phosphate buffered saline (PBS, Sigma-Aldrich, Zwijndrecht, The Netherlands) as control (n = 15). Renal and cremaster perfusion measurements were performed directly after surgical preparation (baseline), 30 min after start of shock induction when a mean arterial pressure (MAP) of 30 mmHg was reached, 1 h after start of shock induction and 0,5, 1, 2, 3 and 4 h after start of fluid resuscitation (Fig. 1a). Blood gas and hematocrit levels were determined at baseline, 1 h after start of shock induction and 1, 2, 3 and 4 h after start of fluid resuscitation.

To exclude a possible effect of microbubbles on the endothelium, a separate group of rats (n = 7) was included in which hemorrhagic shock was induced and only cremaster perfusion measurements were performed (data in supplementary file). Normal saline was given on top of standard fluid resuscitation in comparable volumes as the microbubbles to standardize the protocol.

### Anesthesia and surgical preparation

For surgery, rats were anesthetized with 4% isoflurane (Ivax Farma, Haarlem, The Netherlands) in a plastic box filled with 100% oxygen. Following endotracheal intubation with a 16G catheter (Venflon Pro, Becton Dickinson, Helsingborg, Sweden), lungs were mechanically ventilated throughout the complete experiment (UMV-03, UNO Roestvaststaal BV, Zevenaar, The Netherlands) with a positive end-expiratory pressure of 1–2 cm H_2_O, a respiratory rate of ~ 65 breaths/min, a tidal volume of ~ 10 ml/kg and 1.5–2.0% isoflurane in oxygen-enriched air (40% O_2_/60% N_2_). Respiratory rate was adjusted to maintain pH and partial pressure of carbon dioxide within physiological limits. The body temperature was continuously measured and maintained stable between 36.5 °C and 37.5 °C using a temperature controller (TC-1000 Rat, CWe Inc., United States). A 22G catheter (Venflon Pro, Becton Dickinson, Helsingborg, Sweden) was placed in the caudal (tail) artery for continuous measurements of arterial blood pressure. For cremaster microcirculatory perfusion measurements, the left cremaster muscle was isolated under warm saline superfusion, spread out on a heated platform (34 °C) and covered with gas impermeable plastic film (Saran wrap) as previously described [10, 21, 22]. The right femoral artery was cannulated with a 20G catheter (Arterial Cannula, Becton Dickinson, Helsingborg, Sweden) for blood withdrawal, blood gas analyses (ABL80, radiometer, Copenhagen, Denmark) and hematocrit measurements. The right jugular vein was catheterized with a 22G catheter (Venflon Pro, Becton Dickinson, Helsingborg, Sweden) for administration of the drugs and infusion of Ringer’s lactate and shed blood, as well as infusion of microbubbles for renal perfusion measurements. All catheter insertions were preceded by local application of 1% lidocaine. Fentanyl (1.25–2.50 μg) was administered via the caudal artery for additional analgesia every 20–30 min throughout the experiment. Arterial blood pressure, central venous pressure, ECG and heart rate were continuously recorded using PowerLab software (PowerLab 8/35, Chart 8.0; ADInstruments Pty, Ltd., Castle Hill, Australia).

### Hemorrhagic shock and fluid resuscitation

Hemorrhagic shock was induced as previously described [10] by withdrawing blood with from the right femoral artery until a MAP of 30 mmHg was reached and maintained for 1 h either by withdrawal of blood or reinfusion of heparinized shed blood. Rate of blood withdrawal started at 0.8 ml/min until MAP of 50 mmHg was reached and was subsequently lowered to 0.2 ml/min. After 1 h of hemorrhagic shock, rats were resuscitated with Ringer’s lactate (Fresenius Kabi, Zeist, The Netherlands; 1 × volume of withdrawn blood) and shed blood (preserved in a syringe with 200 UI heparin (LEO Pharma, Amsterdam, The Netherlands)) with a reinfusion rate of 0.7 ml/min until baseline levels of MAP were regained. To mimic the clinical setting, a fixed-dose of 200 ng vasculotide in 100 μl PBS or 100 μl PBS alone as control was administered intravenously between the “pre-hospital” phase (i.e., resuscitation with Ringer’s lactate) and the “in-hospital” phase (i.e., resuscitation with shed blood). Optimal treatment concentration was based on dose–response experiments and previously performed experiments by our group [10, 22] (Additional file 1: Fig. S1).

Rats were monitored for 4 consecutive hours in which MAP was maintained > 50 mmHg by infusion of residual shed blood and subsequently Ringer’s lactate with an infusion rate of 0.2 ml/min. After killing, the right kidney was used for edema formation determined by wet/dry weight ratio. The left kidney was snap-frozen in liquid nitrogen and stored at − 80 °C for further analyses.

### Renal perfusion with contrast echography

Contrast-enhanced echography (CEUS) was performed as previous described [23–25] with a Vevo 2100 Imaging System and MS 250 Nonlinear Contrast Imaging transducer (VisualSonics Inc, Toronto, Canada). Microbubbles were prepared from perfluorobutane gas and stabilized with a monolayer of distearoyl phosphatidylcholine and polyoxyethylene (PEG) stearate. 1,2-Distearoyl-*sn*-glycero-3-phosphocholine (DSPC; Avanti Polar Lipids, Alabama, USA) and polyoxyethylene stearate (PEG40; Sigma, St. Louis, MO, USA) were dissolved in glycerol (10 mg/mL) and sonicated (Decon FS200, Decon Ultrasonics Ltd., Sussex, UK) at 40 kHz in an atmosphere of perfluorobutane (F2 Chemicals Ltd., Lancashire, UK) and vials were shaken in a Vialmix at 4500 rpm (Bristol-Myers Squibb Medical Imaging, Massachusetts, USA). As the gas was dispersed in the aqueous phase, microbubbles were formed, which were stabilized with a self-assembled lipid/surfactant monolayer. A Multisizer 3 Coulter Counter (Beckman Coulter Inc., Miami, FL, USA) was used to measure the particle size distribution as well as the number of particles. Microbubbles with a particle range between 1 and 10 µm were diluted in degassed saline to a concentration of 2.5·10^9^/ml.

For renal perfusion measurements, microbubbles were continuously infused into the jugular vein with a rate of 150 µl/min (during shock with a rate of 100 µl/min due to 30% reduction in circulating volume) using a syringe pump (World Precision Instruments Germany GmbH, Berlin, Germany) at the predefined time points (Fig. 1a). After two minutes of microbubble infusion, perfusion images were taken from the longitudinal plane of the right kidney. A burst of high acoustic power was applied to destroy the microbubbles. Subsequently, images with low acoustic power were acquired to allow contrast replenishment in the kidney. This destruction-replenishment sequence was repeated two times.

Regions of interest were drawn in the renal cortex (Fig. 1d). Signal intensities from the frames after microbubble destruction were corrected for background noise by subtracting the signal intensity of the first frame after microbubble destruction (*Y*0). These intensities were then fitted (*Y* = *Y*0 + (*A* − *Y*0) ⋅ (1 − exp^(−β⋅*x*)^)) for calculation of microvascular blood volume (*A*) and microvascular filling velocity (β), which corresponds to the capillary blood exchange rate. The estimate of perfusion was calculated by the product of *A* and β (Fig. 1e). Analyses were performed by an investigator who was blinded for treatment allocation.

### Microcirculatory cremaster perfusion with intravital microscopy

After stabilization of the exposed cremaster muscle for at least 30 min, cremaster microcirculatory perfusion measurements were performed using a 10 × objective on an intravital microscope (AxiotechVario 100HD, Zeiss, Germany) connected to a digital camera (scA640, Basler, Germany) with a final magnification of 640x, as described previously [10, 21, 22]. Three regions of the microvasculature in the cremaster muscle with adequate perfusion quality were selected during baseline. These predefined regions were followed throughout the experiment at predefined time points (Fig. 1a). For perfusion analyses, two vertical lines were drawn in each video screen. The total amount of capillaries per screen was obtained by averaging the counted capillaries crossing the two vertical lines. These small vessels were categorized as continuously perfused, intermittently perfused (blood flow was arrested at least once or flow was reversed), or non-perfused capillaries (vessels without erythrocytes or non-flowing erythrocytes). Analyses were performed by an investigator who was blinded for treatment allocation.

### Renal edema

Renal tissue was harvested at the end of the experiment under terminal anesthesia. Wet tissue was weighed and dried at 70 °C. After 24 h, dry tissue was weighed and renal wet/dry weight ratio was calculated as estimate relative of tissue water content.

### Plasma and urine analyses

Arterial blood was withdrawn in heparin tubes at baseline and before killing. Blood was centrifuged twice at 4 °C (10 min at 4.000×*g* at 4 °C and 15 min at 10.000×*g* at 4 °C) to obtain platelet-free plasma and stored at − 80 °C. Urine samples were obtained upon killing by aspiration from the bladder with a 25G needle, immediately snap-frozen and stored at − 80 °C. Urine levels of neutrophil gelatinase-associated lipocalin (NGAL) and kidney injury marker-1 (KIM-1) and plasma levels of circulating angiopoietin-1, angiopoietin-2, soluble Tie2 (Cloud-Clone Corporation, Katy, TX, USA), KIM-1 (R&D systems, Minneapolis, Minnesota, USA), NGAL (Bioporto, Hellerup, Denmark) and creatinine (MyBioSource, San Diego, California, USA) were measured with enzyme-linked immunosorbent assay (ELISA) in accordance to the manufacturer. Levels of circulating proteins were corrected for hematocrit values.

### Protein analyses

Frozen kidney tissue was homogenized to obtain cellular protein fractions for Western blot analysis as described previously [10, 25]. Protein expression of NGAL, total Tie2, VE cadherin, RhoA and Rac-1 was analyzed using anti-lipocalin-2 (NGAL; ab63929, Abcam, USA), anti-Tie2 (AF762, R&D systems, USA), anti-RhoA (#2117, Cell Signaling Technology, USA) and anti-Rac-1 (No. 610650, BD Biosciences, USA). Signals were normalized to glyceraldehyde 3-phosphate dehydrogenase (GAPDH; No. 2118, Cell Signaling Technology, USA) for loading control. Immunoblots were quantified by densitometric analysis of films (ImageQuant TL, v8.1, GE Healthcare, USA) by an investigator who was blinded for treatment allocation.

### RNA analyses

Total RNA was extracted from 10–20 mg frozen kidney tissue and isolated using the RNeasy mini kit (Qiagen, Venlo, the Netherlands) as previously described [10, 22]. The RNA concentration and purity were determined using NanoDrop 1000 (NanoDrop Technologies, Wilmington, DE, USA). A total of 1 μg RNA was transcribed into complementary DNA using an iScript cDNA synthesis kit (Bio-Rad, Veenendaal, the Netherlands) using oligo-dT priming. mRNA abundance was measured using a CFX384 Touch real-time PCR detection system (Bio-Rad, Veenendaal, the Netherlands). The following primers were used for quantitative polymerase chain reaction: Tie2, NGAL, KIM-1, ICAM-1, VCAM-1, E-selectin, P-selectin, RhoA and Rac-1 (Applied Biosystems, Foster City, California, USA). ΔΔcT values were calculated and mRNA expression levels were normalized to Arbp abundance (Applied Biosystems, Foster City, California, USA).

### Histology

Four-micrometer-thick paraffin sections were stained with periodic acid–Schiff after diastase [26]. Tubular injury, characterized by necrosis, dilation, cast deposition, and loss of brush border, was graded to the extent of corticomedullary region involvement in 10 randomly chosen, non-overlapping fields (×20 magnification), on a scale from 0 to 4, as follows: 0, absent; 1, 0 to 25%; 2, 25 to 50%; 3, 50 to 75%; 4, 75 to 100%. Total values were expressed as tubular injury scores. The degree of injury was scored by a pathologist specialized in renal pathology who was blinded to group allocation.

### Statistical analysis

Data were expressed as mean ± SD and analyzed using GraphPad Prism 8.0 (GraphPad Software, USA). At least a reduction in renal perfusion from 240 to 139 ± 100 (unit less) and a reduction in cremaster perfusion from 10.1 to 6.9 ± 1.7 perfused vessels per recording after hemorrhagic shock were expected, based on pilot experiments and previously published data [10], respectively. With a two-sided significance level (α) of 0.05 and β of 0.9, group sizes of 13 for renal perfusion measurements and 7 for cremaster perfusion measurements were calculated.

The normality distribution of data was calculated using the Shapiro–Wilk test. For data with a normal distribution, differences between groups were examined for statistical significance using one-way analysis of variance (ANOVA) followed by Bonferroni post hoc analyses for comparison of multiple groups, or Student’s *t* test for comparison between two groups. Time-dependent differences in the characteristics of the hemorrhagic shock model and in cremaster microcirculatory perfusion measurements between groups were analyzed using a two-way ANOVA with repeated measurements, renal perfusion measurements using a two-way ANOVA without repeated measurements, followed by Bonferroni post hoc analyses. For data that were not normally distributed, differences between groups were examined using Kruskal–Wallis test followed by Dunn’s analyses for multiple comparison for comparison of multiple groups, or Mann–Whitney U test for comparison between two groups. *P* values less than 0.05 were considered statistically significant.

## Results

### Hemodynamic values and blood gas analysis

All hemodynamic values and results of blood gas analysis are presented in Table 1.

**Table 1:** Hemodynamics and blood gas values

Parameter	HS				HS + VT			
	Baseline	1 h HS	R + 1 h	R + 4 h	Baseline	1 h HS	R + 1 h	R + 4 h
MAP (mmHg)	85 ± 13	27 ± 5 *	66 ± 14 *	65 ± 9 *	83 ± 9	26 ± 2 *	64 ± 11 *	57 ± 10 *
Heart rate (BPM)	369 ± 39	319 ± 36 *	336 ± 29 *	330 ± 30 *	367 ± 26	304 ± 25 *	342 ± 20 *	333 ± 24 *
Temperature	36.4 ± 0.7	36.0 ± 0.5	36.3 ± 0.5	36.2 ± 0.5	36.7 ± 0.6	36.1 ± 0.8 *	36.3 ± 0.6	36.3 ± 0.5
CVP (mmHg)	1 ± 1	2 ± 1	4 ± 2 *	6 ± 2 *	2 ± 1	2 ± 1	3 ± 1 *	5 ± 1 *
Respiratory rate (min^−1^)	65 ± 5	70 ± 5	75 ± 5 *	75 ± 5 *	65 ± 5	70 ± 5	75 ± 5 *	75 ± 5 *
pH	7.39 ± 0.07	7.30 ± 0.04 *	7.32 ± 0.05 *	7.29 ± 0.08 *	7.39 ± 0.04	7.31 ± 0.04 *	7.34 ± 0.04 *	7.29 ± 0.07 *
pCO_2_ (kPa)	5.47 ± 1.10	5.18 ± 0.81	6.06 ± 0.92	4.88 ± 0.91	5.03 ± 0.69	5.06 ± 0.60	5.57 ± 0.83	5.26 ± 1.33
HCO_3_^−^ (mmol/L)	24.1 ± 1.4	18.5 ± 2.0 *	22.3 ± 1.6 *	17.5 ± 1.2 *	22.7 ± 2.3	19.1 ± 1.8 *	21.8 ± 2.1 *	17.8 ± 3.4 *
Base excess (mEq/L)	− 0.2 ± 1.0	− 6.8 ± 1.7 *	− 3.4 ± 1.5 *	− 7.8 ± 1.3 *	− 1.6 ± 2.0	− 6.5 ± 2.0 *	− 3.4 ± 1.6 *	− 7.4 ± 3.0 *
pO_2_ (kPa)	32.9 ± 8.2	33.7 ± 5.6	33.9 ± 5.1	33.5 ± 4.2	33.5 ± 7.7	33.5 ± 5.4	34.1 ± 5.6	32.1 ± 5.5
sO_2_ (%)	99.7 ± 0.3	99.8 ± 0.1	99.8 ± 0.1	99.8 ± 0.1	99.8 ± 0.3	99.8 ± 0.2	99.8 ± 0.1	99.7 ± 0.2
Hematocrit (%)	40 ± 2	31 ± 2 *	33 ± 2 *	24 ± 6 *	39 ± 4	32 ± 3 *	34 ± 3 *	26 ± 8 *

Data are presented as mean ± SD. * P < 0.05 compared to the baseline value of the corresponding group. No differences were found between vasculotide (VT) and untreated HS rats. HS; hemorrhagic shock group, HS + VT; hemorrhagic shock group treated with vasculotide, 1 h HS; values measured at 1 h following start blood withdrawal, R + 1 h; values measured at 1 h following start of fluid resuscitation, R + 4 h; values measured at 4 h following of start fluid resuscitation, MAP; mean arterial pressure, CVP; central venous pressure,

In accordance with the experimental protocol, induction of hemorrhagic shock decreased MAP (Fig. 1b, p < 0.0001), which was accompanied by a decrease in heart rate (Fig. 1c, p < 0.0001). Fluid resuscitation targeted a MAP > 50 mmHg (*p* < 0.0001) and increased heart rate (*p* < 0.01 vs. 1 h HS), however both values did not restore to baseline values (*p* < 0.0001 R + 0.5 h vs. baseline). Both variables remained unaltered during the follow-up period compared to baseline values (both *p* < 0.0001 vs. baseline).

Hemorrhagic shock decreased pH, bicarbonate and base excess (all values: *p* < 0.0001 vs. baseline). Fluid resuscitation partly restored bicarbonate levels and base excess (both *p* < 0.0001 vs. 1 h HS), but this temporarily restoration in the subsequent hours. pH was not affected by fluid resuscitation and remained altered during the follow-up period (*p* > 0.99 vs. 1 h HS). Hematocrit decreased following hemorrhagic shock and further decreased during fluid resuscitation. pCO_2_, pO_2_, and sO_2_ remained unaltered during the complete experiment (all values: *p* > 0.99). Central venous pressure remained unaltered during hemorrhagic shock (*p* = 0.50 vs. baseline), but increased following fluid resuscitation and in the subsequent hours (*p* < 0.0001 vs. 1 h HS).

Vasculotide treatment did not affect MAP, heart rate, central venous pressure, hematocrit or blood gas values (all values: *p* > 0.99).

### Cremaster perfusion assessed with intravital microscopy

**Fig. 2:**
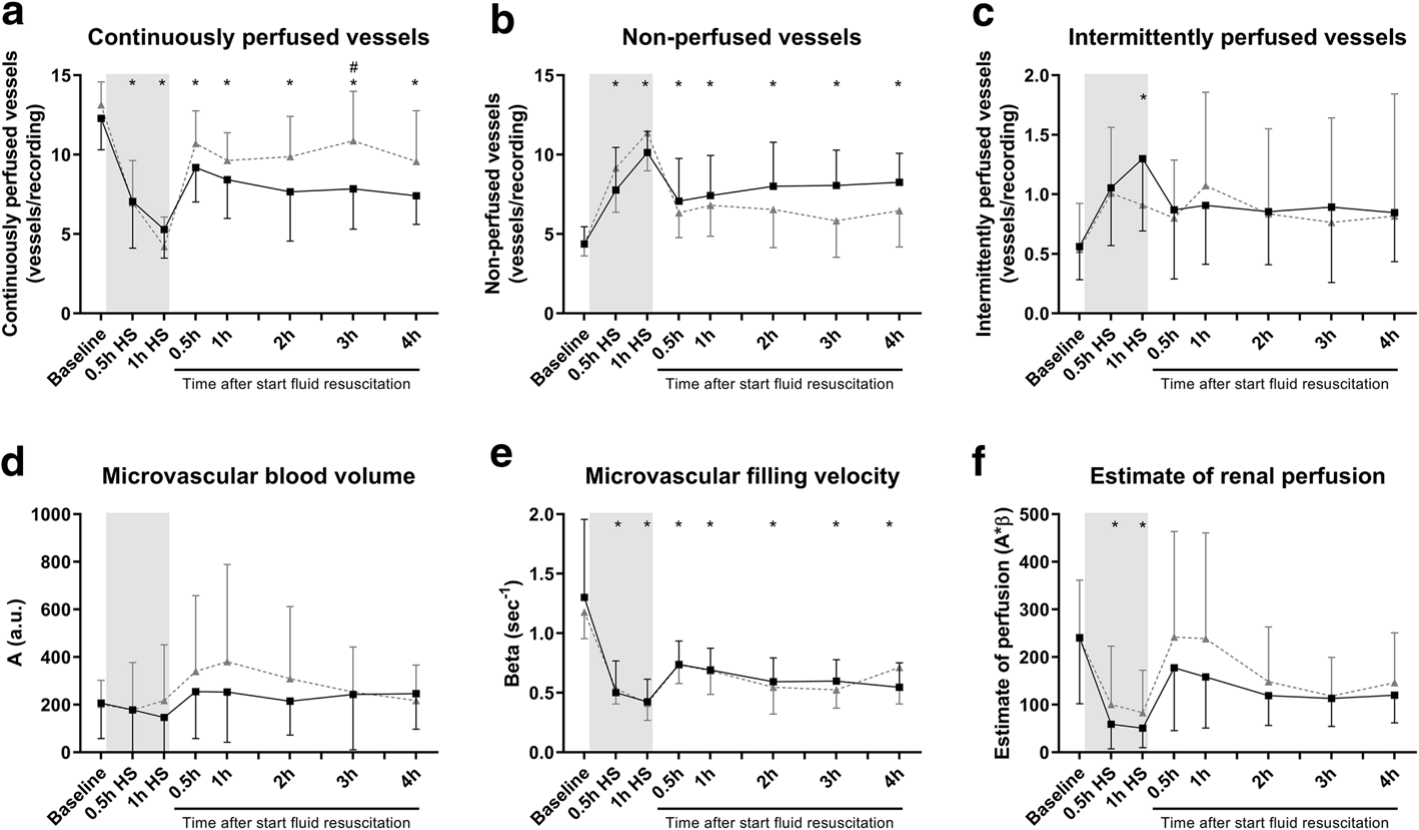
Cremaster and renal perfusion following hemorrhagic shock and fluid resuscitation. Continuously perfused vessels (**a**), non-perfused (**b**) and intermittently perfused vessels (**c**) in rat cremaster muscle using intravital microscopy. Renal microvascular blood volume *A* (**d**), microvascular filling velocity *β* (**e**) and estimate of renal perfusion (A*β; **f**) as assessed by contrast-enhanced ultrasound echography in rats during and after hemorrhagic shock and fluid resuscitation as control (HS; black line) or with vasculotide treatment (HS + VT; grey dotted line). Two-way ANOVA with Bonferroni post hoc analyses, *P < 0.05 HS group compared to baseline; ^#^P < 0.05 HS + VT vs. HS group. Data represent mean ± SD, n = 13

In the cremaster muscle, hemorrhagic shock decreased the number of continuously perfused vessels (Fig. 2a: from 12 ± 2 (baseline) to 5 ± 2 (1 h HS) perfused vessels/recording, *p* < 0.0001) and increased the number of non-perfused vessels (Fig. 2b: from 4 ± 1 (baseline) to 10 ± 1 (1 h HS) non-perfused vessels/recording *p* < 0.0001). Both parameters were partly restored by fluid resuscitation, but did not reach baseline values (R + 0.5 h: 9 ± 2 perfused vessels/recoding *p* = 0.0001; 7 ± 3 non-perfused vessels/recording *p* < 0.0001 vs 1 h HS), and remained stable during the subsequent hours (R + 4 h: 7 ± 2 perfused vessels/recording *p* = 0.10; 8 ± 2 non-perfused vessels/recording *p* = 0.35, vs. R + 0.5 h). Hemorrhagic shock resulted in an increase in intermittently perfused vessels (Fig. 2c: *p* < 0.001, vs. baseline), which was partly corrected by fluid resuscitation (*p* = 0.15, R + 0.5 h vs. 1 h HS) and remained stable during subsequent hours (*p* > 0.99, R + 4 h vs. R + 0.5 h).

Treatment with vasculotide restored cremaster perfusion compared to untreated animals (Table 2). This was reflected by a significant increase in the number of perfused vessels at 3 h following fluid resuscitation (R + 3 h: 11 ± 3 vs 8 ± 3, *p* < 0.05, HS + VT vs. untreated HS), but not at other time points (R + 0.5 *p* > 0.99, R + 1 h *p* > 0.99, R + 2 h *p* = 0.20, R + 4 h *p* = 0.22, vs untreated HS). No significant differences between groups were found in the number of intermittently perfused or non-perfused vessels, but treatment with vasculotide tended to reduce the number of non-perfused vessels at 3 h following start of fluid resuscitation (*p* = 0.11, vs. untreated HS rats).

**Table 2:** Cremaster perfusion and renal perfusion data

Parameter	Group	Time point							
		Baseline	0.5 h HS	1 h HS	R + 0.5 h	R + 1 h	R + 2 h	R + 3 h	R + 4 h
Cremaster perfusion									
Continuously perfused vessels (vessels/recording)	HS	12 ± 2	7 ± 3*	5 ± 2*	9 ± 2*	8 ± 2*	8 ± 3*	8 ± 3*	7 ± 2*
	HS + VT	13 ± 1	7 ± 3*	4 ± 2*	11 ± 2*	10 ± 2*	10 ± 3*	11 ± 3*^	10 ± 3*
Intermittently perfused vessels (vessels/recording)	HS	0.6 ± 0.3	1.0 ± 0.5	1.3 ± 0.6*	0.9 ± 0.6	0.9 ± 0.5	0.9 ± 0.4	0.9 ± 0.6	0.8 ± 0.4
	HS + VT	0.5 ± 0.4	1.0 ± 0.6	0.9 ± 0.4	0.8 ± 0.5	1.0 ± 0.8	0.8 ± 0.7	0.8 ± 0.9	0.8 ± 1.0
Non-perfused vessels (vessels/recording)	HS	4 ± 1	8 ± 3*	10 ± 1*	7 ± 3*	7 ± 3*	8 ± 3*	8 ± 2*	8 ± 2*
	HS + VT	4 ± 1	9 ± 3*	11 ± 2*	6 ± 2*	7 ± 2*	7 ± 2*	6 ± 2*	6 ± 2*
Renal perfusion									
Microvascular blood volume *A* (a.u.)	HS	206 ± 149	178 ± 258	146 ± 149	254 ± 196	253 ± 211	215 ± 142	243 ± 233	246 ± 149
	HS + VT	201 ± 101	178 ± 199	217 ± 234	340 ± 318	380 ± 408	309 ± 302	253 ± 190	217 ± 149
Microvascular filling velocity *B* (s^−1^)	HS	1.3 ± 0.7	0.5 ± 0.3*	0.4 ± 0.2*	0.7 ± 0.2*	0.7 ± 0.8*	0.6 ± 0.2*	0.6 ± 0.2*	0.5 ± 0.2*
	HS + VT	1.2 ± 0.2	0.5 ± 0.1*	0.4 ± 0.1*	0.7 ± 0.2*	0.7 ± 0.2*	0.5 ± 0.2*	0.5 ± 0.2*	0.7 ± 0.3*
Estimate of perfusion	HS	241 ± 139	59 ± 52*	51 ± 41*	177 ± 131	158 ± 107	119 ± 63	113 ± 59	120 ± 58
	HS + VT	234 ± 122	100 ± 122*	83 ± 89*	242 ± 222	238 ± 222	148 ± 115	118 ± 81	146 ± 105

Data presented as mean ± SD. * P < 0.05 compared to baseline value of the corresponding group. ^ P < 0.05 vasculotide effect, compared to the same time point of the untreated HS group. HS; hemorrhagic shock group, HS + VT; hemorrhagic shock group treated with vasculotide. Time points represent 0.5 h following start of blood withdrawal (0.5 h HS), 1 h following start of blood withdrawal (1 h HS) and 0.5 h (R + 0.5 h), 1 h (R + 1 h), 2 h (R + 2 h), 3 h (R + 3 h) and 4 h (R + 4 h) following start of fluid resuscitation

### Renal perfusion assessed with contrast echography

Renal microvascular blood volume (*A*) was not affected by hemorrhagic shock nor fluid resuscitation (Fig. 2d; 246 ± 149 vs 206 ± 149 a.u. *p* > 0.99, R + 4 h vs baseline). Induction of hemorrhagic shock decreased microvascular filling velocity (*β*; Fig. 2e; from baseline: 1.3 ± 0.7 to 1 h HS: 0.4 ± 0.2 s^−1^, *p* < 0.0001), which partly restored by fluid resuscitation (R + 0.5 h: 0.7 ± 0.2 s^−1^ *p* < 0.05) and remained unaltered in the subsequent hours (R + 4 h: 0.5 ± 0.2 s^−1^ *p* > 0.99 vs R + 0.5 h). Estimate of renal perfusion decreased as a result of hemorrhagic shock (Fig. 2f; from baseline: 240 ± 139 to 1 h HS: 40 ± 41, *p* < 0.001). Renal perfusion was only partly restored by fluid resuscitation (R + 0.5 h: 177 ± 131, *p* = 0.07), and remained unaltered in the consecutive hours (R + 4 h: 120 ± 58, *p* > 0.99 vs R + 0.5 h).

Treatment with vasculotide did not affect microvascular blood volume, microvascular filling velocity nor renal perfusion (all *p* > 0.99) compared to untreated HS rats (Table 2).

### Edema formation and fluid requirements

Treatment with vasculotide had no significant effect on renal edema formation as assessed by wet/dry ratios (4.97 ± 0.4 vs. 5.35 ± 0.64, *p* = 0.15). Similar amounts of blood were withdrawn to induce hemorrhagic shock in vasculotide-treated HS animals and untreated HS animals (6.9 ± 1.2 vs. 6.8 ± 1.2 ml; *p* > 0.99). Also, no differences were found in resuscitation fluid volumes to reach baseline values (7.3 ± 2.5 vs. 7.5 ± 2.8 ml; *p* > 0.99) or total resuscitation fluid volume between vasculotide-treated HS animals and untreated HS animals (21.5 ± 11.5 vs. 20.8 ± 8.2 ml; *p* > 0.99).

### Angiopoietin/Tie2 and inflammatory signaling

**Fig. 3:**
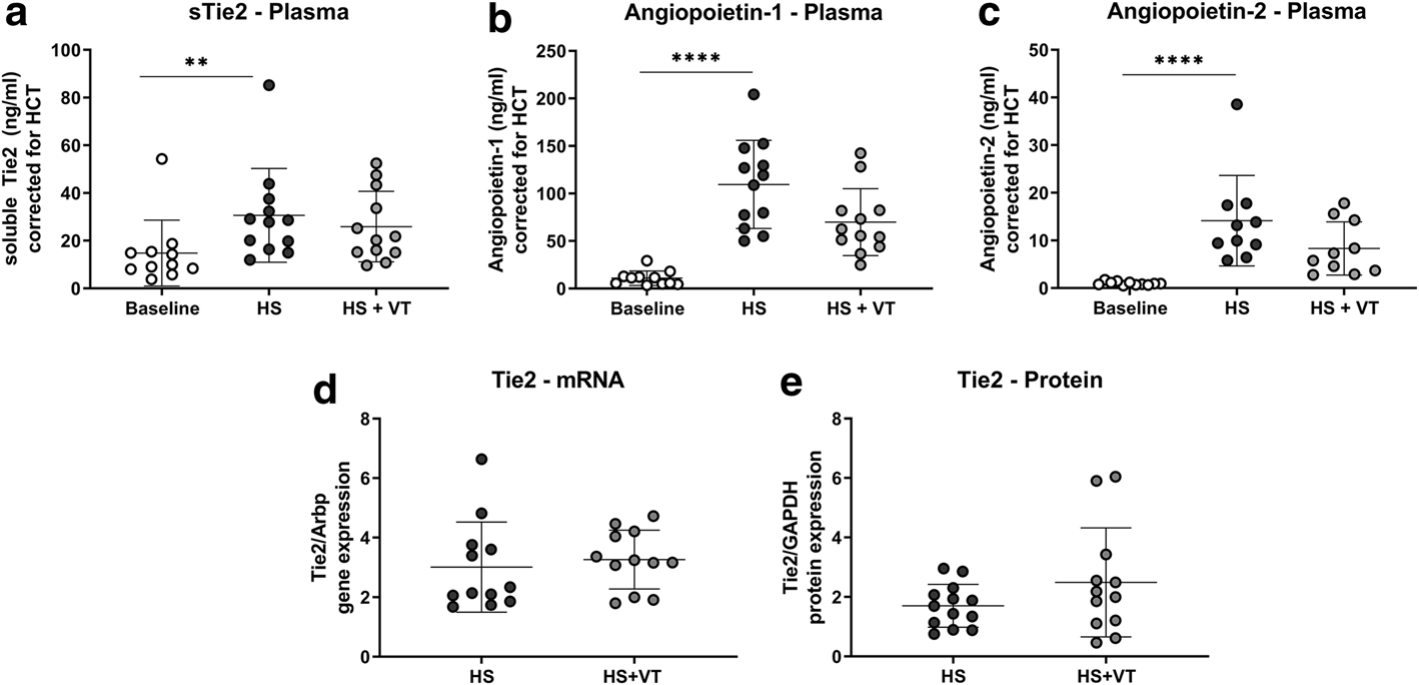
Expression levels of the angiopoietin/Tie2 system. Plasma levels of soluble Tie2 (**a**), angiopoietin-1 (**b**) and angiopoietin-2 (**c**), measured at baseline (white circles) and following hemorrhagic shock and fluid resuscitation (HS; black circles, HS + VT; grey circles). Renal gene expression of Tie2 (**d**) and renal protein expression of total Tie2 (**e**) measured at 4 h after fluid resuscitation (HS; black circles, HS + VT; grey circles). Data represent mean ± SD. Plasma: Kruskal–Wallis with Dunn’s analyses. Gene and protein expression: Student’s T-test. *P < 0.05 HS group compared to baseline; ^#^P < 0.05 HS + VT vs. HS group. VT; vasculotide

Hemorrhagic shock and fluid resuscitation increased circulating soluble Tie2 (Fig. 3a, p < 0.01), angiopoietin-1 (Fig. 3b, p < 0.0001) and angiopoietin-2 (Fig. 3c, p < 0.0001) levels compared to baseline values. Treatment with vasculotide did not affect soluble Tie2 levels (*p* > 0.99), renal Tie2 mRNA levels (Fig. 3d, p = 0.26), renal Tie2 protein expression levels (Fig. 3e, supplemental Fig. 2, *p* = 0.41), circulating angiopoietin-2 levels (*p* = 0.52) or circulating angiopoietin-1 levels (*p* = 0.29) compared to untreated HS rats. Additionally, treatment with vasculotide did not affect mRNA levels of RhoA and Rac-1, downstream targets of the angiopoietin/Tie2 system (Additional file 1: Figs. S2, S3; mRNA expression; Rac-1, *p* = 0.51; RhoA, *p* = 0.58; protein expression; Rac-1, *p* = 0.78; RhoA, *p* = 0.28) compared to untreated animals. Moreover, treatment with vasculotide did not affect renal gene expression of cell endothelial cell adhesion molecules; ICAM-1, VCAM-1, P-selectin and E-selectin (Additional file 1: Fig. S4; ICAM-1, p = 0.59; VCAM-1, *p* = 0.42; E-selectin, *p* = 0.57; P-selectin, *p* = 0.17).

Increased circulating angiopoietin-2 levels positively associated with increased renal edema formation at 4 h after start of resuscitation (r = 0.82, *p* < 0.0001). No relation was found between increased circulating angiopoietin-1 levels and renal edema formation (r = 0.009, *p* = 0.97) or soluble Tie2 levels and renal edema formation (r = 0.18, *p* = 0.42).

### Renal injury

**Fig. 4:**
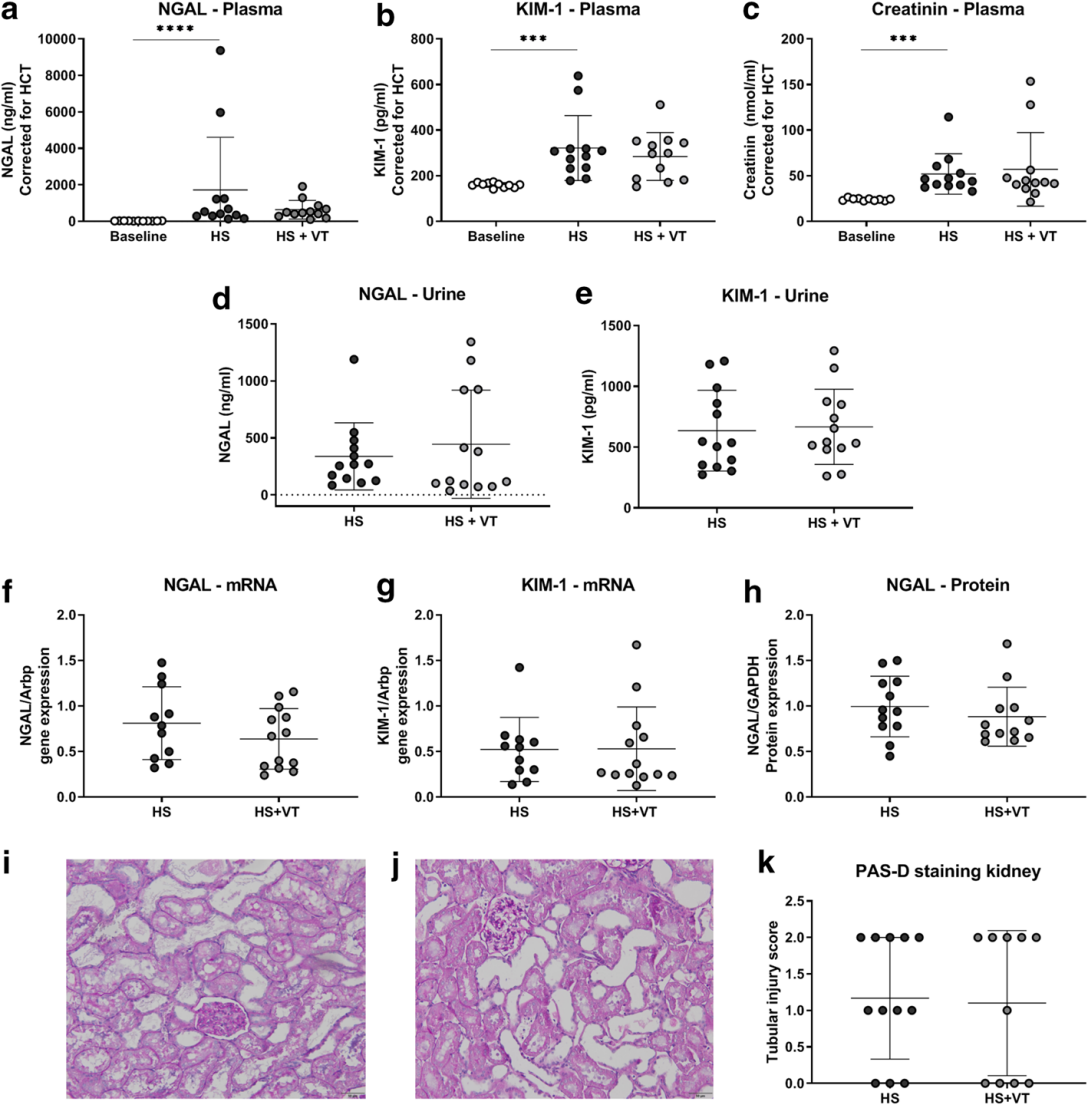
Expression levels of renal injury markers. Plasma levels of NGAL (**a**), KIM-1 (**b**) and creatinine (**c**), measured at baseline (white circles) and following hemorrhagic shock and fluid resuscitation (HS; black circles, HS + VT; grey circles). Urinary levels of NGAL (**d**) and KIM-1 (**e**), measured following hemorrhagic shock and fluid resuscitation (HS; black circles, HS + VT, grey circles). Renal gene expression of NGAL (**f**) and KIM-1 (**g**), and renal protein expression of NGAL (**h**) measured following hemorrhagic shock and fluid resuscitation (HS; black circles, HS + VT; grey circles). Microphotographs (original magnification × 20) of PAS-d stained renal tissue sections from untreated HS rats (**i**) and VT-treated HS rats (**j**), showing similar degrees of ischemic tubular injury, as evidenced by tubular dilation and loss of brush borders, tubular injury scores represented in graph (**k**). Data represent mean ± SD. Plasma: Kruskal–Wallis with Dunn’s analyses. Gene and protein expression: Student’s T-test. *P < 0.05 HS group compared to baseline; # P < 0.05 HS + VT vs. HS group. *VT* vasculotide, *NGAL* neutrophil gelatinase-associated lipocalin, *KIM-1* kidney injury marker-1

Hemorrhagic shock and fluid resuscitation increased circulating NGAL (Fig. 4a, p < 0.05), KIM-1 (Fig. 4b, p < 0.01) and creatinine (Fig. 4c, p < 0.05) levels compared to baseline values. Treatment with vasculotide did not affect circulating NGAL (Fig. 4a, p = 0.27), KIM-1 (Fig. 4b, p = 0.78) or creatinine (Fig. 4c, p > 0.99) levels, nor did it affect urinary levels of NGAL (Fig. 4d, p = 0.50) or KIM-1 (Fig. 4e, p = 0.81) compared to untreated animals. In renal tissue, treatment with vasculotide did not affect mRNA expression levels of NGAL (Fig. 4f, p = 0.26) or KIM-1 (Fig. 4g, p = 0.96), or protein expression levels of NGAL (Fig. 4h, supplemental Fig. 4, *p* = 0.41), measured at 4 h after fluid resuscitation and compared to untreated HS rats. The degree of renal damage was similar in both groups (Fig. 4i–k), as assessed via histopathological analysis.

### Effect of microbubbles

In most variables, no differences were found in hemodynamics (Additional file 1: Fig. S5A–F) and cremaster perfusion (Additional file 1: Fig. S5G, H) between HS rats that did, or did not receive microbubbles. However, rats that did not receive microbubbles had increased levels of bicarbonate at 2 h after starting fluid resuscitation (22.4 ± 0.4 vs 19.7 ± 1.4 mmol/L, *p* < 0.01) and increased levels of base excess at 2 h (− 3.4 ± 1.4 vs − 5.4 ± 1.4 mEq/L, *p* < 0.05) and 3 h after starting fluid resuscitation (− 3.5 ± 1.8 vs − 6.0 ± 1.0 mEq/L, *p* < 0.01) compared to HS rats that received microbubbles. However, these differences abolished over time as at 4 h after starting fluid resuscitation no differences were observed. Also, no differences in renal wet/dry ratios were found between HS control rats receiving microbubbles and HS control rats that did not receive microbubbles (Additional file 1: Fig. S5I, p = 0.14).

## Discussion

In the present study, we showed that hemorrhagic shock impairs renal and cremaster perfusion. The clinical standard therapy with fluid resuscitation only partly restored renal and cremaster perfusion, but could not restore perfusion back to baseline values in both organs. These perfusion disturbances were paralleled by renal injury and an imbalance in key components of the endothelial angiopoietin/Tie2 system. Interestingly, the proposed relation between elevated angiopoietin-2 levels and organ failure was confirmed in the current study, as increased circulating angiopoietin-2 levels were associated with increased renal edema formation. However, treatment with the Tie2 agonist vasculotide did not improve renal perfusion, nor did it reduce renal injury. Treatment with vasculotide did temporarily improve cremaster perfusion during fluid resuscitation, indicating biological activity of the compound. Our data suggest that stimulation of Tie2 is not a suitable approach following hemorrhagic shock and fluid resuscitation to improve renal perfusion and function.

Previously, we showed that hemorrhagic shock impaired microcirculatory perfusion in the cremaster muscle in rats, which was accompanied by increased pulmonary and renal microvascular leakage [10]. In the present study, we confirmed the detrimental effects of hemorrhagic shock on cremaster microcirculatory perfusion. Additionally, we showed that hemorrhagic shock impaired renal perfusion. Although the available knowledge regarding renal perfusion following hemorrhagic shock is limited, our data are in line with previously performed animal studies. Hemorrhagic shock reduced cortical renal perfusion as determined by IDF microscopy [9] and blood loss > 35% of circulating volume deteriorated renal perfusion as measured by invasive laser Doppler flowmetry [27]. Here, we show the applicability of a non-invasive imaging technique, contrast-enhanced ultrasonography, which can be used in the clinical setting to determine hemorrhagic shock-induced renal perfusion disturbances [28, 29]. Importantly, the effect of hemorrhagic shock on microcirculatory perfusion in the present study was tissue-specific as renal perfusion was more severely affected by hemorrhagic shock than cremaster perfusion with a reduction in perfusion of 80% versus 57%, respectively, compared to baseline values. This confirms the vulnerability of the renal vasculature after hemorrhagic shock [9, 30, 31] and highlights the importance of selective imaging of perfusion in vital organs and the need for interventions that restore kidney perfusion.

To date, general treatment options following hemorrhagic shock aim to restore circulating volume by fluid resuscitation. However, fluid overload can lead to multiple organ failure and worsen outcome [32]. In the current study, we showed that with a non-aggressive fluid resuscitation strategy by targeting MAP > 50 mmHg, renal perfusion is not restored back to baseline levels after HS. Current transfusion protocols target a specific MAP, however, the optimal target MAP is still up for debate [33, 34]. Our study clearly shows that restoration of MAP with a permissive fluid resuscitation strategy does not restore renal perfusion. A hypotensive resuscitation strategy targeting a MAP of 50 mmHg reduces transfusion requirements compared to a target MAP of 65 mmHg [35, 36] and improves survival among trauma patients [37], whereas aggressive fluid resuscitation is known to induce cardiac and pulmonary complications [38]. In contrast, renal perfusion and glomerular filtration appear pressure-dependent below a MAP of 75 mmHg, leading to reduced renal oxygenation [39], which might explain the lack of restoration of renal perfusion during fluid resuscitation. In conclusion, we showed that standard fluid resuscitation failed to restore renal and cremaster perfusion, confirming that additional treatment strategies are warranted.

One of the underlying pathological features that may explain the discrepancy between effects on global hemodynamics and tissue perfusion is microvascular leakage. Following hemorrhagic shock and fluid resuscitation, microvascular leakage increases in both kidney and lungs [10]. Especially during fluid resuscitation, microvascular leakage has detrimental effects as it reduces the benefits of fluid therapy [40]. As a consequence of endothelial hyperpermeability, fluids can partially leak to the interstitium, leading to tissue edema. Tissue edema increases diffusion distances, which impairs oxygen delivery and may eventually lead to organ failure as result of tissue hypoperfusion. The angiopoietin/Tie2 system is a key regulator of endothelial permeability. Following hemorrhagic shock and fluid resuscitation, the angiopoietin/Tie2 signaling changes extensively [8, 10, 17], which is also confirmed in the current study. This detrimental shift towards angiopoietin-2/Tie2 signaling is associated with the development of AKI following trauma [15] or cardiac surgery [16], and with negative clinical outcome [15]. Elevated angiopoietin-2 levels have previously been associated with in vitro endothelial hyperpermeability [11, 41]. In line with this observation, we observed that elevated angiopoietin-2 levels were associated with renal edema formation, proposing angiopoietin-2 as contributor to or interesting biomarker for the development of kidney injury following hemorrhagic shock. Collectively, we confirmed the angiopoietin/Tie2 system as potential target to reduce microvascular leakage.

In the current study, we targeted Tie2 with vasculotide to reduce microvascular leakage and thereby improve the effectiveness of fluid resuscitation, as reflected by improvement of microcirculatory perfusion and renal function. Previously performed studies reported the capability of vasculotide to enhance Tie2 phosphorylation, as determined in rat glomerular endothelial cells [10] and septic mice [43]. Here, we found that treatment with vasculotide temporarily restored cremaster perfusion, but did not affect renal perfusion or renal injury. One possible explanation for this may be the organ-specific expression levels of Tie2, as Tie2 has a relatively low expression pattern in renal tissue compared to pulmonary tissue [42]. Several studies investigated the effect of vasculotide in diverse animal models of critical illness [19, 43, 44]. In septic mice, vasculotide reduced microvascular leakage in lungs [43, 44], but not in kidneys or in the spleen [44]. In contrast with our findings, Rübig et al. reported an improvement in renal perfusion and function following treatment with vasculotide in mice with renal ischemia–reperfusion injury [19]. Differences in outcome may be a function of injury severity, disease model, host animal or timing and dosage of treatment. Unfortunately, increasing the dosage of vasculotide resulted in adverse effects on microcirculatory perfusion as determined in pilot experiments, as reflected by suboptimal microcirculatory perfusion in sham animals. This dose-dependent effect was also confirmed by Kümpers et al., as an increased dosage of vasculotide reduced survival following sepsis compared to a lower dosage [43]. Taken together, treatment with vasculotide was unable to restore renal perfusion and function in the current set-up.

*Strengths and limitations *The current study is one of the first to assess microcirculatory perfusion in different organs within the same animal due to the use of two imaging techniques. This resulted in the interesting and important finding that the given treatment showed organ-specific efficiency. Second, although renal injury is a known complication following hemorrhagic shock and fluid resuscitation, knowledge regarding the course of renal perfusion during and following hemorrhagic shock was lacking. However, the current study assessed renal injury in a fairly short time frame. Even though all circulating renal injury markers confirmed the development of renal injury, a possible beneficial effect of vasculotide over a longer period cannot be excluded. As there are no specific antibodies available to assess Tie2 phosphorylation in rat kidney tissue, it was not possible to measure activation of the Tie2 receptor following vasculotide treatment. However, previous studies confirmed the capability of vasculotide to enhance Tie2 phosphorylation in mice [43] and rat glomerular cell culture [10].

### Conclusion

Hemorrhagic shock attenuated renal and cremaster perfusion in rats, and perfusion in both organs was only partly restored following fluid resuscitation. These perfusion disturbances were paralleled by renal injury and an imbalance in key components of the angiopoietin/Tie2 system. Targeting Tie2 using vasculotide did not improve renal perfusion or function, but temporarily restored cremaster perfusion. Our results show that targeting Tie2 using vasculotide is not an appropriate strategy to restore renal perfusion disturbances following hemorrhagic shock. Future studies should focus on targeting the angiopoietin/Tie2 system via a different approach, such as decreasing circulating angiopoietin-2 levels, or could investigate the effect of activation of Tie2 in other affected organs to improve organ failure other than the kidneys.

## Supplementary Information

**Additional file1**:** Figure S1.** Vasculotide dose-dependent efficiency on cremaster perfusion in sham and hemorrhagic shock rats. **Figure S2.** Raw data of western blot analysis. **Figure S3.** Expression levels of markers reflecting endothelial barrier function. **Figure S4.** Expression levels of cell adhesion molecules. Figure S5. Effect of microbubbles on hemodynamic values, cremaster perfusion and renal wet/dry ratio.

## Data Availability

The datasets used and/or analyzed during the current study are available from the corresponding author on reasonable request.

## Abbreviations

AKI: Acute kidney injury
ANOVA: Analysis of variance
CEUS: Contrast-enhanced echography
DSPC: Distearoyl phosphatidylcholine
ELISA: Enzyme-linked immunosorbent assay
GAPDH: Glyceraldehyde 3-phosphate dehydrogenase
HS: Hemorrhagic shock
ICAM-1: Intercellular adhesion molecule 1
MAP: Mean arterial pressure
KIM-1: Kidney injury marker-1
NGAL: Neutrophil gelatinase-associated lipocalin
PAS-D: Periodic acid–Schiff after diastase
PBS: Phosphate buffered saline
PEG: Polyoxyethylene
VCAM-1: Vascular cell adhesion protein 1
VT: Vasculotide

## References

[1] Hutchings SD, Naumann DN, Hopkins P (2018). Microcirculatory impairment is associated with multiple organ dysfunction following traumatic hemorrhagic shock: The MICROSHOCK Study. Crit Care Med.

[2] Harrois A, Libert N, Duranteau J (2017). Acute kidney injury in trauma patients. Curr Opin Crit Care.

[3] Harrois A, Soyer B, Gauss T (2018). Prevalence and risk factors for acute kidney injury among trauma patients: a multicenter cohort study. Crit Care.

[4] Tachon G, Harrois A, Tanaka S (2014). Microcirculatory alterations in traumatic hemorrhagic shock. Crit Care Med.

[5] Verma SK, Molitoris BA (2015). Renal endothelial injury and microvascular dysfunction in acute kidney injury. Semin Nephrol.

[6] Legrand M, Mik EG, Balestra GM (2010). Fluid resuscitation does not improve renal oxygenation during hemorrhagic shock in rats. Anesthesiology.

[7] Aksu U, Demirci C, Ince C (2011). The pathogenesis of acute kidney injury and the toxic triangle of oxygen, reactive oxygen species and nitric oxide. Contrib Nephrol.

[8] Yan R, van Meurs M, Popa ER (2019). Early heterogenic response of renal microvasculature to hemorrhagic shock/resuscitation and the influence of NF-κB pathway blockade. Shock.

[9] Eguillor JFC, Ferrara G, Edul VSK (2020). Effects of Systemic Hypothermia on Microcirculation in Conditions of Hemodynamic Stability and in Hemorrhagic Shock. Shock.

[10] Trieu M, van Meurs M, van Leeuwen ALI (2018). Vasculotide, an angiopoietin-1 mimetic, restores microcirculatory perfusion and microvascular leakage and decreases fluid resuscitation requirements in hemorrhagic shock. Anesthesiology.

[11] van Leeuwen ALI, Naumann DN, Dekker NAM (2020). In vitro endothelial hyperpermeability occurs early following traumatic hemorrhagic shock. Clin Hemorheol Microcirc.

[12] Sack KD, Kellum JA, Parikh SM (2020). The angiopoietin-Tie2 pathway in critical illness. Crit Care Clin.

[13] Roviezzo F, Tsigkos S, Kotanidou A (2005). Angiopoietin-2 causes inflammation in vivo by promoting vascular leakage. J Pharmacol Exp Ther.

[14] Richter RP, Russell RT, Hu PJ (2019). Plasma angiopoietin-2/-1 ratio is elevated and angiopoietin-2 levels correlate with plasma syndecan-1 following pediatric trauma. Shock.

[15] Ganter MT, Cohen MJ, Brohi K (2008). Angiopoietin-2, marker and mediator of endothelial activation with prognostic significance early after trauma?. Ann Surg.

[16] Jongman RM, Van Klarenbosch J, Molema G (2015). Angiopoietin/Tie2 dysbalance is associated with acute kidney injury after cardiac surgery assisted by cardiopulmonary bypass. PLoS ONE.

[17] van Meurs M, Kurniati NF, Wulfert FM (2009). Shock-induced stress induced loss of microvascular endothelial Tie2 in the kidney which is not associated with reduced glomerular barrier function. Am J Physiol Renal Physiol.

[18] Ghosh CC, David S, Zhang R (2016). Gene control of tyrosine kinase TIE2 and vascular manifestations of infections. Proc Natl Acad Sci U S A.

[19] Rübig E, Stypmann J, Van Slyke P (2016). The synthetic Tie2 agonist peptide vasculotide protects renal vascular barrier function in experimental acute kidney injury. Sci Rep.

[20] Reynolds P, Wall P, van Griensven M (2012). Shock supports the use of animal research reporting guidelines. Shock.

[21] Koning NJ, de Lange F, van Meurs M (2018). Reduction of vascular leakage by imatinib is associated with preserved microcirculatory perfusion and reduced renal injury in a rat model of cardiopulmonary bypass. Br J Anaesth.

[22] Dekker NAM, van Meurs M, van Leeuwen ALI (2018). Vasculotide, an angiopoietin-1 mimetic reduces pulmonary vascular leakage and preserves microcirculatory perfusion during cardiopulmonary bypass in rats. Br J Anaesth.

[23] Boly CA, Eringa EC, Bouwman RA (2016). The effect of perioperative insulin treatment on cardiodepression in mild adiposity in mice. Cardiovasc Diabetol.

[24] Van den Brom CE, Boly CA, Bulte CSE (2016). Myocardial perfusion and function are distinctly altered by sevoflurane anesthesia in diet-induced prediabetic rats. J Diabetes Res.

[25] van den Brom CE, Bulte CS, Kloeze BM (2012). High fat diet-induced glucose intolerance impairs myocardial function, but not myocardial perfusion during hyperaemia: a pilot study. Cardiovasc Diabetol.

[26] Roelofs JJ, Rouschop KM, Leemans JC (2006). Tissue-type plasminogen activator modulates inflammatory responses and renal function in ischemia reperfusion injury. J Am Soc Nephrol.

[27] Erni D, Banic A, Wheatley AM (1995). Haemorrhage during anaesthesia and surgery: continuous measurement of microcirculatory blood flow in the kidney, liver, skin and skeletal muscle. Eur J Anaesthesiol.

[28] Malhi H, Grant EG, Duddalwar V (2014). Contrast-enhanced ultrasound of the liver and kidney. Radiol Clin North Am.

[29] Lin Q, Lv F, Luo Y (2001). (2015) Contrast-enhanced ultrasound for evaluation of renal trauma during acute hemorrhagic shock: a canine model. J Med Ultrason.

[30] Sutton TA, Fisher CJ, Molitoris BA (2002). Microvascular endothelial injury and dysfunction during ischemic acute renal failure. Kidney Int.

[31] Torres Filho I (2017). Hemorrhagic shock and the microvasculature. Compr Physiol.

[32] Claure-Del Granado R, Mehta RL (2016). Fluid overload in the ICU: evaluation and management. BMC Nephrol.

[33] Forni LG, Joannidis M (2017). Blood pressure deficits in acute kidney injury: not all about the mean arterial pressure?. Crit Care.

[34] Sato R, Luthe SK, Nasu M (2017). Blood pressure and acute kidney injury. Crit Care.

[35] Carrick MM, Leonard J, Slone DS (2016). Hypotensive resuscitation among trauma patients. Biomed Res Int.

[36] Morrison CA, Carrick MM, Norman MA (2011). Hypotensive resuscitation strategy reduces transfusion requirements and severe postoperative coagulopathy in trauma patients with hemorrhagic shock: preliminary results of a randomized controlled trial. J Trauma.

[37] Albreiki M, Voegeli D (2018). Permissive hypotensive resuscitation in adult patients with traumatic haemorrhagic shock: a systematic review. Eur J Trauma Emerg Surg.

[38] Cotton BA, Guy JS, Morris JA (2006). The cellular, metabolic, and systemic consequences of aggressive fluid resuscitation strategies. Shock.

[39] Skytte Larsson J, Bragadottir G, Redfors B (2018). Renal effects of norepinephrine-induced variations in mean arterial pressure after liver transplantation: a randomized cross-over trial. Acta Anaesthesiol Scand.

[40] Dilken O, Ergin B, Ince C (2020). Assessment of sublingual microcirculation in critically ill patients: consensus and debate. Ann Transl Med.

[41] Dekker NAM, van Leeuwen ALI, van Strien WWJ (2019). Microcirculatory perfusion disturbances following cardiac surgery with cardiopulmonary bypass are associated with in vitro endothelial hyperpermeability and increased angiopoietin-2 levels. Crit Care.

[42] Aslan A, van Meurs M, Moser J (2017). Organ-specific differences in endothelial permeability-regulating molecular responses in mouse and human sepsis. Shock.

[43] Kumpers P, Gueler F, David S (2011). The synthetic tie2 agonist peptide vasculotide protects against vascular leakage and reduces mortality in murine abdominal sepsis. Crit Care.

[44] David S, Ghosh CC, Kümpers P (2011). Effects of a synthetic PEG-ylated Tie-2 agonist peptide on endotoxemic lung injury and mortality. Am J Physiol Lung Cell Mol Physiol.

