# Supplementary material for: The effect of targeting Tie2 on hemorrhagic shock-induced renal perfusion disturbances in rats
Source: Intensive Care Med Exp. 2021 May 17;9:23. doi: 10.1186/s40635-021-00389-5 (PMC8126531; doi:10.1186/s40635-021-00389-5)
Supplement: Supplementary file 1 — Additional file1: Figure S1. Vasculotide dose-dependent efficiency on cremaster perfusion in sham and hemorrhagic shock rats. Figure S2. Raw data of western blot analysis. Figure S3. Expression levels of markers reflecting endothelial barrier function. Figure S4. Expression levels of cell adhesion molecules. Figure S5. Effect of microbubbles on hemodynamic values, cremaster perfusion and renal wet/dry ratio. [file 40635_2021_389_MOESM1_ESM.docx]

**The effect of targeting Tie2 on hemorrhagic shock-induced renal perfusion disturbances in rats**

# Supplemental Digital Content

Anoek L.I. van Leeuwen^1,2,3^, Nicole A.M. Dekker^1,2,3^, Paul Van Slyke^4^, Esther de Groot^1^, Marc G. Vervloet^5^, Joris J.T.H. Roelofs^6^, Matijs van Meurs^7,8^, Charissa E. van den Brom^1,2^

^1^Department of Anesthesiology, Experimental Laboratory for Vital Signs, Amsterdam Cardiovascular Sciences, Amsterdam UMC, Vrije Universiteit, Amsterdam, The Netherlands;

^2^Department of Physiology, Amsterdam Cardiovascular Sciences, Amsterdam UMC, Vrije Universiteit, Amsterdam, The Netherlands;

^3^Department of Cardiothoracic Surgery, Amsterdam Cardiovascular Sciences, Amsterdam UMC, Vrije Universiteit, Amsterdam, The Netherlands;

^4^Vasomune Therapeutics, Toronto, Canada;

^5^Department of Nephrology, Amsterdam Cardiovascular Sciences, VU University Medical Center, Amsterdam, the Netherlands;

^6^Department of Pathology, Amsterdam Cardiovascular Sciences , Academic Medical Center, University of Amsterdam, Amsterdam, Netherlands;

^7^Department of Pathology and Medical Biology, Medical Biology section, University Medical Center Groningen, Groningen, The Netherlands;

^8^Department of Critical Care Medicine, University Medical Center Groningen, Groningen, The Netherlands;

^9^Department of Intensive Care, Amsterdam UMC, University of Amsterdam, Amsterdam, the Netherlands

**SUPPLEMENTAL FIGURE 1**: Vasculotide dose-dependent efficiency on cremaster perfusion in sham and hemorrhagic shock rats.

**SUPPLEMENTAL FIGURE** **2**: Raw data of western blot analysis.

**SUPPLEMENTAL FIGURE 3**: Expression levels of markers reflecting endothelial barrier function.

**SUPPLEMENTAL FIGURE** **4**: Expression levels of cell adhesion molecules.

**SUPPLEMENTAL FIGURE 5**: Effect of microbubbles on hemodynamic values, cremaster perfusion and renal wet/dry ratio.

**SUPPLEMENTAL FIGURE 1:** Vasculotide dose-dependent efficiency on cremaster perfusion in sham and hemorrhagic shock rats, as performed prior to the study published by Trieu *et al*, 2018. Details regarding the experimental set-up have been published previously (Trieu et al, Anesthesiology 2018;128(2):361-374). The effect of different doses of vasculotide was determined in the cremaster muscle using intravital microscopy (n = 2-3 per group). In sham setting, rats received either one of the three different doses (200 ng, 350 ng and 500 ng) or PBS as placebo. The 500 ng dose (n=1) immediately resulted in a severe disturbance of microcirculatory perfusion and was therefore excluded for further assessment. The remaining two doses (200 ng and 350 ng) were tested in a model of hemorrhagic shock and fluid resuscitation (HS+R).


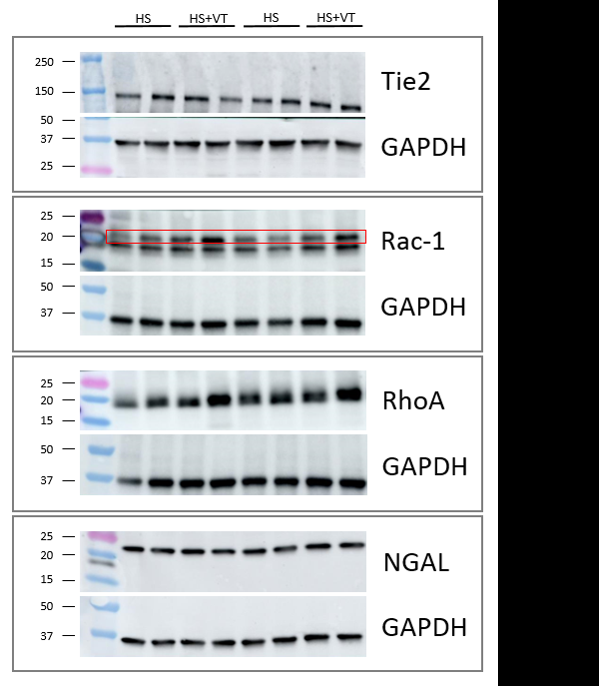


**SUPPLEMENTAL FIGURE** **2**: Raw data of western blot analysis. Renal protein expression levels were assessed using antibodies against Tie2, Rac-1, RhoA and neutrophil gelatinase-associated lipocalin (NGAL). For all analysis, signals were normalized to glyceraldehyde 3-phosphate dehydrogenase (GAPDH). Images represent typical examples of tissues from rats that underwent 1 hour of hemorrhagic shock followed by 4 hours of fluid resuscitation without additional treatment (HS) or rats that underwent the same procedure but received treatment with vasculotide (HS+VT).

**SUPPLEMENTAL FIGURE 3**: Expression levels of markers reflecting endothelial barrier function. Renal gene expression levels of Rac-1 (**A**) and RhoA (**B**) and protein expression levels of Rac-1 (**C**) and RhoA (**D**), measured following hemorrhagic shock and 4h of fluid resuscitation (HS; black circles, HS+VT, grey circles). Gene expression levels were normalized to Arbp gene expression, protein expression levels were normalized to GAPDH expression. Data represent mean ± SD.

** SUPPLEMENTAL FIGURE** **4**: Cell adhesion molecules. Renal gene expression of intercellular adhesion molecule 1 (ICAM-1; **A**), vascular cell adhesion molecule 1 (VCAM-1; **B**), E-selectin (**C**) and P-selectin (**D**) was determined in renal tissue of rats following hemorrhagic shock and fluid resuscitation (HS), treated with either vasculotide (VT; grey circles) or phosphate buffered saline (PBS; black circles) as control. Gene expression levels were normalized to Arbp gene expression. Data represent mean ± SD.

#

# SUPPLEMENTAL FIGURE 5: Effect of microbubbles on hemodynamic values, cremaster perfusion and renal wet/dry ratio. Mean arterial pressure (A), heart rate (B), hematocrit (C), pH (D), bicarbonate (E), base excess (F) levels were assessed at baseline, during 1h of hemorrhagic shock and in the 4 hours following fluid resuscitation in rats. Cremaster perfusion was assessed predetermined time points using intravital microscopy (G & H). Renal wet/dry ratios were determined after sacrifice as measure of renal edema formation (I). Rats either received PBS and underwent contrast enhanced ultrasound with microbubble infusion (squares with continuous line) or underwent the same experimental protocol without microbubble infusion (triangles with dotted line). Two-way ANOVA with Bonferroni post hoc analyses, *P < 0.05 HS group compared to baseline; $ P < 0.05 HS – no microbubbles vs. HS group. Data represent mean ± SD, n = 13 (HS), n = 7 (HS – no microbubbles).
